# Inclusive Art Programs for Dementia Families: National Taiwan Museum of Fine Arts

**DOI:** 10.1002/alz70858_102627

**Published:** 2025-12-26

**Authors:** Tsuann Kuo

**Affiliations:** ^1^ Chung Shan Medical University, Taichung City, Taiwan, Taiwan

## Abstract

Taiwan has implemented its Dementia Action Plan 2.0 for 8 years and Inclusive Art Programs have been developed after 6 years of preparation. The “Arts Together for Wise Friends,” an effort coordinated by the research staff from the Taiwan National Museum of Fine Arts, provided dementia friendly art tour to dementia families. This creation was based on the concept of dementia friendly and equity for people with disabilities. Every two months, a trained docent, with the accompany of 3‐4 trained college students from Chung Shan Medical University (CSMU), provided a 1.5‐hour art tour to 2‐3 dementia families. The activities included the appreciation of art pieces and art history, along with group discussion about the time spent together. This purpose of this paper was to evaluate the outcomes of this inclusive art program for the dementia families and those who involved. The qualitative research method was used by doing detailed interviews to those participants, including the students, the docents, and people with dementia and their families. A total of 10 people were interviewed: 4 dementia families, 3 docents and 3 students. Theme analysis was conducted and four major categories were grouped: resilience in dementia families, respite and relaxation, empathy and communication, road to dementia friendliness. The dementia families grew closer in relationship after attending the event while docents and students were able to develop their communication and problem solving skills. This paper concluded by providing critical elements to make inclusive art programs successful and to provide implications for future program develop and policy agenda to create a dementia friendly society.

